# Bioprospecting of the Phylum Bacteroidota for Sustainable Agriculture

**DOI:** 10.3390/plants15101500

**Published:** 2026-05-14

**Authors:** José Luis Ávila-Oviedo, Vicente Montejano-Ramírez, Francisco Javier Campos-Mendoza, Eduardo Valencia-Cantero

**Affiliations:** Instituto de Investigaciones Químico Biológicas, Universidad Michoacana de San Nicolás de Hidalgo, Edifico B1, Ciudad Universitaria, Morelia 58030, Michoacán, Mexico; 1355646g@umich.mx (J.L.Á.-O.); 0678380c@umich.mx (V.M.-R.); 1700143a@umich.mx (F.J.C.-M.)

**Keywords:** Bacteroidota, plant–microbe interactions, nutrient cycling, sustainable agriculture, plant growth promotion, stress tolerance

## Abstract

Global population growth poses major challenges to agricultural systems, demanding more efficient strategies to secure food production. Conventional approaches have relied heavily on chemical inputs; however, their overuse disrupts ecosystems, threatens biodiversity, and undermines human and environmental health. To ensure sustainable productivity, it is essential to explore alternative approaches that leverage microbial functions to enhance plant growth and resilience. Bacteria are among the most abundant soil microorganisms, playing central roles in biogeochemical cycles and plant health. While well-studied phyla such as Pseudomonadota, Actinomycetota, and Bacillota have been widely applied as biofertilizers and biocontrol agents, members of the phylum Bacteroidota remain comparatively understudied despite being consistently abundant in plant-associated microbiomes. This review synthesizes current knowledge on Bacteroidota, highlighting their taxonomy, ecological diversity, contributions to nutrient cycling, and mechanisms that promote plant growth, as well as biotic and abiotic stress tolerance. We also discuss the limitations that hinder their application, particularly challenges in cultivation and isolation, and outline future research directions to harness their potential for sustainable agriculture.

## 1. Introduction

The global agricultural sector is under increasing pressure due to the rapid growth of the human population, which requires the intensification of food production systems [1]. Historically, chemical fertilizers and pesticides have been widely used to maintain yields; however, their indiscriminate application has led to soil degradation, ecosystem pollution, biodiversity loss, and risks to human health [2]. These challenges underscore the urgent need for sustainable and environmentally friendly alternatives that safeguard food security without compromising ecosystem integrity.

Agroecological practices such as intercropping, agroforestry, crop–livestock integration, soil conservation, and crop diversification—represent one pathway toward sustainable intensification [3]. Another promising strategy is the use of beneficial microorganisms, including filamentous fungi [4,5], yeasts [6,7], protozoa [8,9], and bacteria [10,11], many of which have been developed as biofertilizers or biopesticides, including globally used formulations based on *Bacillus*, *Pseudomonas*, and *Streptomyces* species for the control of fungal and bacterial plant pathogens [12].

Bacteria are often dominant members of soil microbial communities and are integral to biogeochemical cycles of carbon, nitrogen, and phosphorus, making them essential to ecosystem balance [13,14,15]. The bacterial community plays a key role in plant fitness and resilience [16]. Their association with plants confers multiple benefits through both direct and indirect mechanisms. Direct mechanisms include solubilization and mobilization of nutrients such as phosphorus [17], potassium, nitrogen, and iron, as well as the production of phytohormones such as gibberellic acid (GA), abscisic acid (ABA), cytokinins (CKs), indole-3-acetic acid (IAA), and volatile organic compounds [18,19,20]. Indirect mechanisms include antibiosis via volatile and diffusible secondary metabolites [19,20,21,22], enzymatic activities such as ACC deaminase [23], and the activation of plant defense pathways through elicitor molecules like lipopolysaccharides (LPS) [24] and other extracellular compounds, such as lipopeptides, polyketides, and dipeptides [25]. These traits, however, are often strain-dependent and not universally distributed among bacterial taxa.

Plant-associated bacterial communities vary across compartments of rhizosphere, endosphere, and phyllosphere and their composition is influenced by soil type, pH, moisture, altitude, temperature, plant genotype, and developmental stage [26,27]. Across these environments, Pseudomonadota, Actinomycetota, Bacillota, Acidobacteriota, Verrucomicrobiota, and Bacteroidota are consistently reported as dominant phyla [28,29,30,31]. Several genera, including *Pseudomonas*, *Azospirillum*, *Azotobacter*, *Streptomyces*, *Bacillus*, *Paenibacillus*, *Enterobacter*, *Serratia*, *Burkholderia*, *Herbaspirillum*, *Stenotrophomonas*, and *Rhizobium*, are already commercialized as bioinoculants due to their beneficial properties [32,33,34,35,36]. In contrast, the phylum Bacteroidota has received comparatively little attention despite its consistent abundance and ecological relevance in plant-associated microbiomes.

Despite these advances, limitations persist in the application of microbial products. Their efficacy is often influenced by environmental conditions, product shelf-life, competition with native microbes, and compatibility with host plants [37,38,39]. Furthermore, cultivation-based studies capture only a fraction of microbial diversity, as it is estimated that only 2–25% of soil bacterial taxa can be cultured in the laboratory using standard techniques [40,41]. This knowledge gap is particularly relevant for Bacteroidota, a phylum frequently detected as abundant in plant microbiomes but still poorly studied in depth [16,28,29,42]. The limited knowledge stems largely from challenges associated with their isolation and cultivation [43,44,45].

While numerous studies have focused on well-established plant-beneficial bacteria, Bacteroidota remain underexplored, particularly regarding their functional roles and biotechnological potential. This gap underscores the need for a targeted and integrative analysis of this phylum. Therefore, this review synthesizes current knowledge on the taxonomy, ecological roles, and plant-associated functions of Bacteroidota, emphasizing their contributions to nutrient cycling, plant growth promotion, and stress tolerance. Furthermore, it highlights the disconnect between their ecological relevance and limited agricultural application, outlining key research directions for their integration into microbiome-based sustainable agriculture.

## 2. Taxonomy and Diversity

Members of the phylum Bacteroidota are Gram-negative bacteria broadly distributed across diverse ecosystems, including freshwater, marine, and terrestrial habitats in temperate, tropical, and polar regions [46,47,48]. They exhibit substantial morphological diversity, with some classes producing carotenoid or flexirubin pigments that confer orange, yellow, red, or pink coloration, while others remain non-pigmented [49,50,51,52]. Cells are non-spore-forming and often exhibit gliding motility. Colony morphology is typically circular, convex, or semi-convex, with entire or wavy margins [53]. Cell morphology ranges from filamentous to coccoid forms with rounded or tapered ends. In 90% of species, the length ranges from 1 to 15 µm and the width from 0.3 to 1.15 µm. Genomic characteristics further reflect high diversity within the phylum, with GC content ranging from 31.5% to 53.1% and genome sizes ranging from 2.6 to 7.1 Mb [54], indicating considerable genomic heterogeneity.

The taxonomy of Bacteroidota has undergone several revisions. The phylum was formed by grouping several apparently dissimilar genera under the *Cytophaga–Flavobacteria–Bacteroides* complex (phylum CFB) [55,56]. The phylum was later referred to as *Bacteroidetes* [53] and subsequently as Bacteroidaota [57], and with new rules, the International Committee on Systematics of Prokaryotes formally corrected the nomenclature to Bacteroidota in 2021 [58].

**Figure 1:**
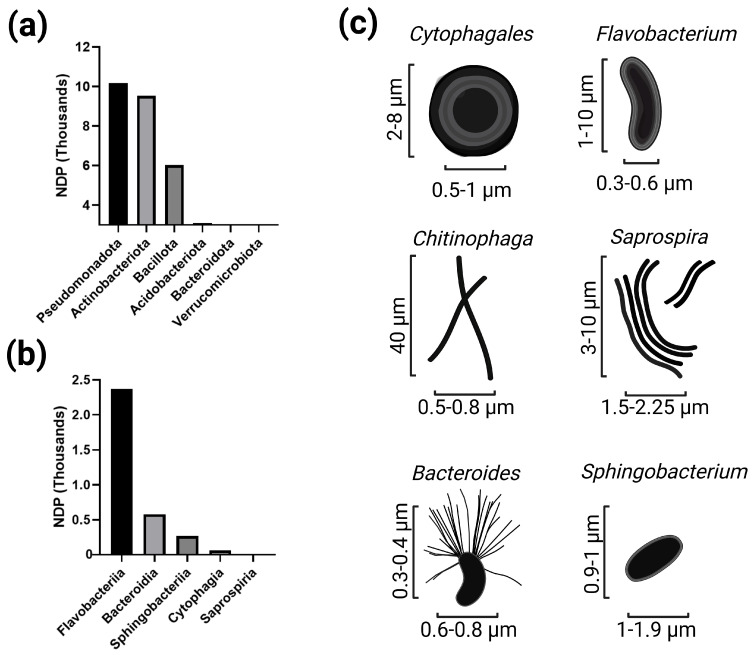
Research trends and morphological diversity of the phylum Bacteroidota. (**a**) Number of published documents (NDP, in thousands) for major plant-associated bacterial phyla, based on the search query “Phylum AND Plant” in the Scopus database (accessed in March 2026). (**b**) Distribution of NDP across classes within the phylum Bacteroidota, based on the search query “class AND plants” in the Scopus database (accessed in March 2026). (**c**) Schematic representation of cell morphology and size ranges of representative genera within the phylum Bacteroidota. Bars indicate approximate cell length and width (µm).

Bacteroidota exhibits considerable taxonomic diversity at the class level, with a total of 136,048 genomes available in public databases. Within this phylum, Bacteroidia is the most abundant class, comprising 96,345 genomes. The other classes include Flavobacteriia with 19,909 genomes, Chitinophagia with 4636, Cytophagia with 3538, Sphingobacteriia with 2284, and Saprospiria with 1996 genomes (NCBI, accessed from https://www.ncbi.nlm.nih.gov/datasets/taxonomy/976/, accessed on 29 April 2026). To date, approximately 3.0 million prokaryotic genomes have been described [59]. Compared with other dominant plant-associated phyla, such as Pseudomonadota and Bacillota, with 1.7 and 0.69 million published genomes, respectively, the approximately 0.13 million genomes attributed to Bacteroidota remain relatively limited [59]. Similarly, research on Actinomycetota and Bacillota in plant-associated contexts far exceeds that on Bacteroidota. Within this phylum, Flavobacteriia and Bacteroidia are relatively well studied, whereas Cytophagia, Sphingobacteriia, Chitinophagia, and Saprospiria remain underrepresented in the literature [60] (Figure 1a,b).

A major bottleneck in the study of Bacteroidota is their limited cultivability, which constrains efforts to explore their functional diversity and biotechnological potential. Conventional isolation strategies typically rely on variations in incubation temperature, pH, growth periods, and nutrient composition, including media such as marine agar [61]. However, conventional cultivation approaches capture only a limited fraction of Bacteroidota diversity.

So-called “Culturomics” involves optimizing growth conditions and high-throughput workflows, utilizing robotics, automation, and next-generation sequencing to identify target microbes of interest. Culturomics-based approaches enable their application to complex plant root microbiomes under field conditions and improve the recovery of a broader diversity of Bacteroidota [62]. These methodologies have been more extensively developed in the human gut microbiome, where their integration with multi-omics strategies has enabled the large-scale isolation of previously uncultured taxa and the functional validation of metagenomic predictions [63,64,65,66].

In contrast, similar integrative efforts remain comparatively underexplored in plant-associated microbiomes. Evidence indicates that the successful isolation of Bacteroidota is strongly dependent on cultivation parameters, including plant compartment, substrate composition, and oxygen availability, with genera such as *Sphingobacterium* and *Chitinophaga* exhibiting selective growth under specific conditions [67]. Furthermore, cultivability is method-dependent, as some taxa are preferentially recovered using advanced strategies, whereas others are more frequently isolated through conventional methods [67].

**Figure 2:**
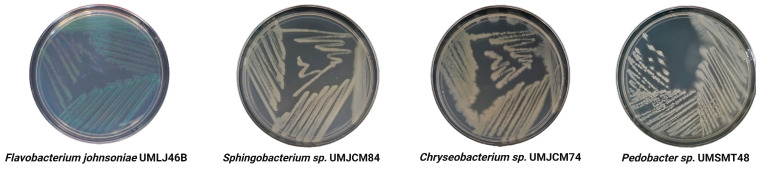
General appearance of *Flavobacterium*, *Sphingobacterium*, *Chryseobacterium* and *Pedobacter* cultured on Reasoner’s 2A. Colony colors include cyan, yellow, orange, and cream. Note the presence of halos of secondary growth from the bacterial colonies, illustrating the gliding motility typical of Bacteroidota.

The most commonly isolated genera of Bacteroidota, when using suitable culture media like Reasoner’s 2A (R2A) and traditional techniques, include *Flavobacterium*, *Sphingobacterium*, *Chryseobacterium* and *Pedobacter* (Figure 2). This focus on specific genera introduces a bias in the study of Bacteroidota toward these microorganisms and underscores the need for advanced cultivation strategies (Table 1). Importantly, even advanced culturomics approaches remain influenced by cultivation conditions, particularly culture media composition, enriching certain microbial groups [65] while underrepresenting others [68]. This inherent selectivity highlights the need for complementary and targeted strategies to better capture the diversity of this phylum.

Accordingly, advanced cultivation frameworks integrating high-throughput culturomics, microfluidic platforms, single-cell techniques, and targeted isolation approaches, often supported by metagenomics and co-culture systems are becoming essential to enhance microbial recovery. These methodologies, including droplet-based cultivation, microfluidic chip systems, and cell-sorting techniques based on functional or phylogenetic traits, have significantly expanded the range of cultivable microorganisms [64]. While the use of these technologies is beginning to spread in the study of the human gut microbiome, their application to plant-associated microbiomes remains limited, highlighting the need to adapt these strategies to better capture the diversity and functional potential of taxa such as Bacteroidota and to fully exploit their value in bioprospecting. Table 1 shows some strategies that have successfully led to the isolation of Bacteroidota, including examples of unconventional approaches.

## 3. Functional Traits

Microbial communities regulate global biogeochemical cycles, with bacteria driving key processes of carbon, nitrogen, and phosphorus turnover in terrestrial and aquatic ecosystems [70,71]. Bacteroidota play an important ecological role due to their strong capacity to degrade complex polysaccharides and other polymers, thereby facilitating nutrient release and recycling [80,81]. In particular, Bacteroidota are ecologically important in agricultural systems, as they contribute to the cycling of nutrients that often act as limiting factors for crop production [82].

### 3.1. Carbon Cycling

Polysaccharides such as hemicelluloses and xyloglucan are major components of plant biomass. Even in monocots, which have much lower levels of these components, they are secreted as root mucilage exudates and contribute to organic matter accumulation and soil fertility [83,84,85]. Bacteroidota are well known for their ability to degrade polysaccharides [60]. They encode a diverse array of carbohydrate-active enzymes, including polysaccharide lyases, carbohydrate esterases, and carbohydrate-binding modules, which contribute to the degradation of complex carbohydrates and thereby influence carbon cycling [86,87].

In aquatic environments, Bacteroidota can degrade algal polysaccharides through xylanolytic, chitinolytic, and alginate and laminarin-degrading activities [88,89]. This metabolic capacity underpins their ecological role as key recyclers of complex organic matter across diverse environments. A study filtering 702 genomes from Flavobacteriales and Cytophagales revealed 100,445 carbohydrate-active enzymes (CAZymes) underscoring the strong polysaccharide-degrading potential of Bacteroidota. Glycoside hydrolases and glycosyltransferases were the most abundant classes, followed by carbohydrate-binding modules, carbohydrate esterases, polysaccharide lyases, and auxiliary activities. Cytophagales exhibited higher CAZyme diversity and abundance than Flavobacteriales, while differences between terrestrial and aquatic strains were reduced after genome-size normalization, suggesting that phylogeny is a stronger determinant of CAZyme repertoires than environmental origin [61].

However, these inferences are largely based on genomic annotations, and the presence of CAZyme-encoding genes does not necessarily reflect their expression or ecological relevance under natural conditions. In addition, current genomic datasets are biased toward cultivable and well-represented taxa, particularly within Flavobacteriia, potentially skewing interpretations of functional potential across the phylum.

This extensive CAZyme repertoire is not randomly distributed in the genome but is typically organized into polysaccharide utilization loci (PULs). In Bacteroidota, these loci represent discrete and co-regulated gene clusters that encode the coordinated machinery required for glycan sensing, binding, depolymerization, and uptake [86]. Genomic and biochemical studies show that, functionally, PULs act as modular systems in which distinct carbohydrate degradation pathways are encoded by specific PUL repertoires shaped by environmental conditions and the structural complexity of target polysaccharides [90,91,92]. Comparative genomic and metagenomic analyses indicate that terrestrial Bacteroidota have specific genetic characteristics and plasticity that enable them to adapt to diverse environments, making them one of the most abundant phyla in soil [93,94].

Microorganisms are essential to leaf litter decomposition; among them, genera such as *Pedobacter* and *Mucilaginibacter* have been identified as key bacteria in nutrient cycling through the decomposition of complex polysaccharides [94]. Using tools such as genome analysis, candidate genes in Bacteroidota involved in carbon metabolism, including glycoside hydrolases, xylosidases, xylanases, arabinanases, arabinofuranosidases, and galactosidases, have been identified [95].

In this way, it is inferred that ecological success is strongly linked to their extensive repertoire of CAZymes, which enables the degradation of complex plant- and fungal-derived polysaccharides.

Comparative metagenomic analyses of soil and rhizosphere microbiomes have revealed that Bacteroidota successfully compete by degrading proteins, plant cell wall polymers, and root mucilage exudate as xyloglucan [96]. There is a consistent enrichment of glycoside hydrolases and PULs targeting plant-derived glycans, particularly xyloglucan, highlighting a clear signature of niche adaptation to hemicellulose-rich environments [97]. For instance, members of the genera *Flavobacterium*, *Pedobacter*, and *Mucilaginibacter* are recurrently associated with the decomposition of plant biomass and leaf litter, reflecting specialization toward structurally complex polysaccharides [94,97]. In addition to CAZyme diversity, experimental and genomic evidence shows that Bacteroidota possess the Type IX Secretion System (T9SS) (described in Section 4.2). T9SS allows protein secretion, and thus enzymatic activity and enhances substrate accessibility. The system facilitates adherence to plant surfaces, efficient colonization, and degradation of complex substrates [98,99,100]. Bacteroidota taxa are enriched in rhizosphere soils relative to bulk soils, reflecting their specialization in utilizing complex plant-derived macromolecules [101].

Using metagenomic approaches, it has been shown that Bacteroidota are present in different compartments of plants (rhizosphere, endosphere, and phyllosphere) [29,102,103], indicating their importance for plant development. Using an experimental approach, it has been found that the fungal endosymbiont *Chitinophaga* sp. PS-EHB01 degrades chitin and utilizes other important compounds in plant-microbe interactions, such as D-trehalose, myo-inositol, and sucrose, thereby affecting the availability of these substrates to other microbial organisms [104].

Beyond substrate degradation, emerging evidence indicates that Bacteroidota contribute to microbial community dynamics through metabolite exchange and cross-feeding interactions. The partial depolymerization of complex substrates, such as chitin or plant-derived glycans, releases nutrients and small chitin oligomers generates oligosaccharides and low-molecular-weight compounds that can be utilized by other microorganisms, promoting a “niche facilitation” effect within the rhizosphere [97,105]. With a culture-dependent approach, it has been shown that *Bacillus cereus* stimulates the growth of *F. johnsoniae* CI04 and other Bacteroidota in the rhizosphere of soybean through a mechanism linked to the metabolization of the *B. cereus* peptidoglycan cell wall [106].

Similarly, metagenomic analyses have found that adding chitin to soil leads to a large, significant increase in Sphingobacteriaceae, among other Bacteroidota [107]; furthermore, assimilation of chitine has been demonstrated by amending ^13^C-chitin laid on a wheat-covered field, the predominantly ^13^C-labeled bacterial populations correspond to Bacillota and uncultured Bacteroidota registering much higher ^13^C level compared with other phyla, principally during the later stages of chitin decomposition under anoxic conditions [108]. Soil and peat substrates amended with chitin increase lettuce growth, accompanied by consistent increases in populations of *Pedobacter*, *Dyadobacter*, and *Arachidicoccus* as determined by 16S amplicon sequencing analysis; plant growth promotion is at least partially explained by chitin degradation to N-acetylglucosamine chitin mixed in the pot and subsequent ammonium release [109,110]. In a different assay, the inoculation of *F. limnicola* strains ST-82^T^, ST-10, and ST-92 in sediments resulted in an increase in protease activity levels 3 to 5-fold compared with uninoculated controls, while approximately 70–80% of the total dissolved nitrogen released was converted to ammonium [111].

### 3.2. Bacteroidota Also Contribute to Nitrogen Transformations

Members of the Bacteroidota are primarily recognized as heterotrophic degraders of complex organic matter, playing an indirect but relevant role in nitrogen cycling [97,105,112]. In line with this ecological role, their main contribution to nitrogen cycling is indirectly mediated through organic matter turnover and interactions with other functional microbial groups, by alternative pathways to nitrate and N_2_O reduction, and, in some cases, nitrogen fixation.

Consistent with these roles, several ecological studies using metagenomic tools indicate that Bacteroidota respond positively to nitrogen availability in soil systems. For instance, experimental designs with different nitrogen fertilization treatments in soil sown with *Triticum aestivum* showed that high doses of inorganic nitrogen at four or ten weeks increased the abundance of several Bacteroidota genera, including *Filimonas*, *Flavisolibacter*, and *Segetibacter* [113]. Studies of wheat nitrogen fertilization over a 10-year period confirm a positive relationship between nitrogen use and Bacteroidota richness [114]. In the same way, continuous cultivation of the nitrogen-fixing legume *Caragana korshinskii* in sandy soil produces soil nitrogen accumulation and an increase in populations of nitrogen-fixing bacteria, such as *Rhizobium*, *Ensifer*, *Neorhizobium*, *Mesorhizobium* (Pseudomonadota), but also *Flavobacterium* and *Chitinophaga* [115]. In laboratory experiments, the genera *Bacteroides* and *Porphyromonas* (Bacteroidota), as well as *Dialister* and *Anaerococcus* (Bacillota), were the dominant microorganisms in a biological oxidation reactor treating ammonium-rich wastewater [116].

Although there is no solid evidence that Bacteroidota oxidize ammonium through canonical nitrification pathways linked to genes *amoABC*, *hao*, and *nxrAB*, commonly considered as ammonium oxidation functional markers that are almost exclusively traditional ammonia-oxidizing archaea, ammonia-oxidizing bacteria, or complete ammonia oxidizer *Nitrospira* [117,118]. Bacteroidota is most likely to play a primarily heterotrophic: degraders and structural maintainers of the microbial communities through niche facilitation effects [97,105], which could indirectly benefit the true nitrifiers.

Biochemical studies with type strains in pure cultures reveal that nitrate reduction to nitrite, or nitrite reduction to molecular nitrogen are often present in typical soil-borne species as *F. denitrificans* ED5C^T^, *F. glaciei* JCM-13953^T^, *F. daejeonense* KACC-11422^T^, *F. banpakuense* 15F3^T^, *F. chungbukense* CS-100^T^, and *F. banpakuense* KACC-14225^T^ but not in *F. johnsoniae* KACC-11410^T^, *F. anhuiense* KCTC-22128^T^, *F. soli* DS-6^T^, *F. tegetincola* ACAM-602^T^, or *F. antarcticum* KCTC-12222^T^ [71,119]. Most of the *Sphingobacterium* strains are nitrate reduction negative, but *S. mizutae* ATCC-33299^T^, and other strains, reduce nitrite to nitrogen gas [120], whereas neither nitrate nor nitrite reduction is found in *Pedobacter* [121,122]. This and other works indicate that classical denitrification is not a distinctive trait of Bacteroidota.

Consistently, metagenomic work in soil shows that key genes for denitrification, such as *napA*, *narG* (coding for nitrate reductases), *nirS*, and *nirK* (coding for nitrite reductases), and *norB* (coding for nitric oxide reductase), are not specifically associated with, or are even absent from, Bacteroidota populations, whereas *nosZ* (codifying for nitrous oxide reductase) is characteristic of this phylum [123,124,125,126]. This finding is relevant because a significant proportion of denitrifying bacteria and archaea produce N_2_O a greenhouse gas, as a terminal product due to the absence of *nosZ* [127]. Accordingly, studies in ammonium-rich removal systems have shown that *Aequorivita* and *Moheibacter* (Bacteroidota), which possess the *nosZ* gene, reduce N_2_O [128,129]. Phan et al. (2025) [126] found that in ammonia recovery bioreactors, Bacteroidota was the most abundant and active phylum. Among the 98 metagenome-assembled genomes (MAGs) from 11 bacterial phyla, 39 MAGs carried *nosZ* genes, including 22 MAGs belonging to Bacteroidota. Among these, *Cloacibacterium* spp. were identified as major N_2_O sinks. Other Bacteroidota such as *Paludibacter*, drive nitrate respiration to ammonium through the Dissimilatory Nitrate Reduction to Ammonium (DNRA) which involves the expression of *qnorB* and *nrfA* genes [126]. It was also shown that approximately one-third of the MAGs with nitrate respiration-related genes possess the *nrfA* gene and that this group mainly comprised Bacteroidota (62%).

Both nitrous oxide reduction and DNRA have great environmental importance and biotechnological potential, since N_2_O reduction prevents the release of this potent greenhouse gas and DNRA allows the recovery of nitrogen from nitrates in the form of ammonium, under carbon-rich and microoxic environments, conditions that would otherwise lead to nitrogen loss through denitrification. Accordingly, *Cloacibacterium* spp. harbor the *cnorB* and *qnor* genes but no nitrate or nitrite reductase genes and are emerging as major promising players for N_2_O conversion to N_2_ due to their fitness to survive and thrive in soil [129,130]. *Cloacibacterium*, *Paludibacter*, and other members of the Bacteroidetes class possess *nosZ* and *nrfA* genes, which facilitate nitrogen reduction through distinct pathways. This highlights the necessity for further research into the regulation of the transition from N_2_O reduction to ammonification metabolism. Such understanding is crucial for practical applications in the field.

### 3.3. Phosphorus Cycling

Phosphorus is a key but often limiting macronutrient in terrestrial and marine ecosystems [131,132]. Microbial processes are essential to phosphorus turnover and mobilization within the soil matrix. Pseudomonadota, Bacillota, Actinomycetota, and Bacteroidota bacteria are frequently involved in phosphorus cycling through the solubilization of inorganic phosphates and the mineralization of organic phosphorus pools [133,134,135,136]. Bacteroidota strains from the genera *Flavobacterium* [134,137,138], *Arachidicoccus* [139], *Chitinophaga* [140,141], *Chryseobacterium*, *Dyadobacter*, and *Niabella* [136] among others, are described as inorganic phosphorus-solubilization bacteria (PSBs).

Bacteria also possess alkaline (LPs) and acid phosphatases (ACP), including PhoA, PhoX, and PhoD, and AcpA, PhoC [142,143,144]; the expression and activity of these phosphatases are repressed by phosphate availability. PafA phosphatase is prevalent in Bacteroideta bacteria; it is highly active toward phosphomonoesters and is insensitive to excess phosphate [145]. PafA phosphatase was initially identified in *Chryseobacterium meningosepticum* (now *Elizabethkingia meningoseptica*) [146]. PafA contributes to the stronger phosphatase activity found in plant-associated *Flavobacterium*, which has been shown to be greater than that of other plant-associated bacteria [147]. The *pafA* gene is widespread in Bacteroidota from plant rhizospheres, soil, gut, and ocean microbiomes, belonging to the classes Flavobacteriia, Cytophagia, Sphingobacteriia, Chitinophagia, and Bacteroidia [142,145], revealing that PafA is an important enzyme in global phosphate cycling with potential applications in sustainable agriculture.

## 4. Plant–Bacteroidota Interactions

### 4.1. Nutrient Availability Increase to Plants

We have already discussed the role of Bacteroidota in degrading complex organic compounds and the concomitant recycling of nutrients [80,81]. In particular, the transformation and recycling of nitrogen and phosphate in open soil and rhizosphere directly affect the availability of these nutrients to plants [123,124,125,126,142,143,144] and, therefore, promote plant growth. We now discuss other aspects of the interaction between plants and bacteria of the phylum Bacteroidota.

### 4.2. Rhizosphere Colonization

Bacteroidota are increasingly recognized as efficient colonizers of plant surfaces, supported by their unique physiological and genetic traits [148,149,150]. A central mechanism is their Type IX Secretion System (T9SS), which allows gliding motility and the secretion of hydrolytic enzymes; this system is also crucial for chitin degradation, resistance to bacteriophages, and S-layer formation, and not only enables colonization but also competition for space [100,151,152].

Gliding motility allows Bacteroidota to move rapidly over surfaces without pili or flagella [153]. It has been established that the core genes involved in the T9SS are also required for gliding motility [100]. Using *F. johnsoniae* as a model, many components of the motility machinery were identified. Twelve *gld* genes are needed for gliding, seven *spr* genes are essential to produce spreading colonies, while *rem* genes encode proteins with functions in cell motility. Gld, Spr, and Rem proteins are thought to be involved in T9SS required to export motility adhesins across the outer membrane [153].

**Figure 3:**
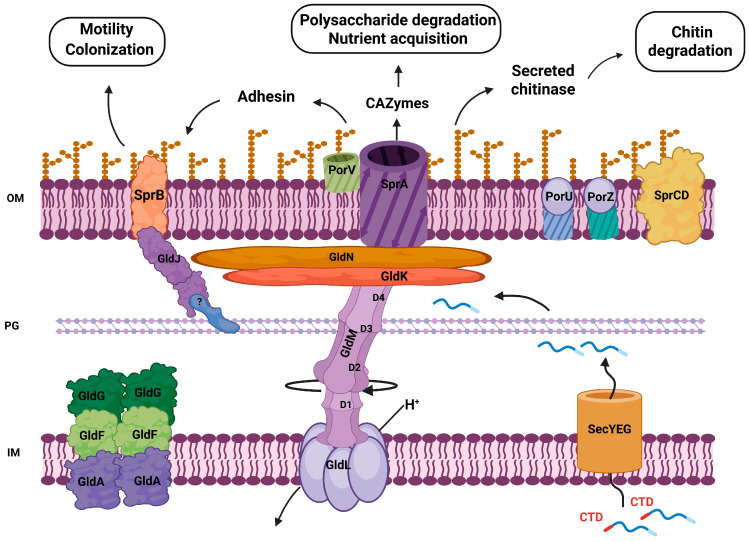
Schematic representation of the Type IX secretion system (T9SS). The system spans the inner membrane (IM), periplasmic space containing peptidoglycan (PG), and outer membrane (OM). Proteins are initially translocated across the inner membrane via the SecYEG complex and targeted to the T9SS through a conserved C-terminal domain (CTD). The inner membrane proteins GldL and GldM form a proton-driven motor that uses the proton motive force (H^+^) to energize the system. The periplasmic domains of GldM (D1–D4) are involved in energy transduction from the inner membrane to the outer membrane. The core structural components GldK and GldN assemble into a ring-like complex that connects the motor to the secretion machinery. Outer membrane proteins, including SprA, function as the secretion pore, while PorV, PorU, and PorZ are involved in substrate translocation, CTD processing, and cell surface attachment of secreted proteins. The SprCD complex contributes to the stability and efficiency of the secretion system. Cytoplasmic and inner membrane-associated proteins GldA, GldF, and GldG form an ABC transporter-like complex involved in motility and system assembly, while GldJ is required for proper assembly and stabilization of the secretion machinery. Surface adhesins such as SprB are secreted and mobilized along the cell surface, enabling gliding motility. Secreted enzymes, including CAZymes and chitinases, participate in the degradation of complex polysaccharides and chitin, contributing to nutrient acquisition and bacterial colonization in plant-associated environments. The question mark indicates a protein with unresolved or not yet fully characterized identity within the T9SS complex.

The general mechanism in *F. johnsoniae* is composed of the proteins GldL, GldM, GldN, GldK, GldJ, SprA, SprE, SprT, PorV, and SprF principally [153,154,155] (Figure 3). GldL forms a proton channel in the inner membrane that powers the rotation of the periplasmic GldM, which, in turn, drives the rotation of a disk formed by GldK and GldN proteins. Torque generation by the GldKN complex enables T9SS function and the displacement of the outer membrane adhesins RemA and SprB [154,156]. RemA and SprB have a lectin domain that binds exopolysaccharides, and it is therefore suggested that it is associated with the binding of plant glycans and, therefore, with motility on the surface of cells [154,156,157].

It has been demonstrated that *Capnocytophaga ochracea* and *F. johnsoniae*, mutants deficient in T9SS components, were unable to produce robust biofilm and lost surface adhesins and gliding motility [158,159]. Biofilm is a key mechanism for attachment and colonization of rhizospheric microenvironments. It has also been shown that Bacteroidota performed a stronger adherence to roots than other bacterial groups [148], but mutations in T9SS components in *Flavobacterium* strongly reduce rhizosphere colonization and persistence, efficiency, and diminish biocontrol activity against pathogens such as the Actinomycota *Clavibacter michiganensis* [160].

These traits explain the consistent presence of Bacteroidota in the rhizosphere, phyllosphere, and endosphere, with higher abundance during early plant growth stages, suggesting a key role in initial root and shoot development [28,161,162,163]. The principal functional traits are summarized in Table 2.

### 4.3. Phytohormone Modulation

Phytohormones play a central role in stress responses and growth regulation. Bacteroidota, like other bacteria, are involved in modulating levels of phytoregulators, either by directly producing these compounds or modifying the plant’s endogenous phytorregulator synthesis.

IAA, the principal auxin in plants, is a key phytohormone that coordinates plant growth; IAA was identified as a growth-promoting phytohormone because it stimulates differential growth in response to light and is involved in multiple physiological and developmental processes [169,170]. Among Bacteroidota, it is documented that several genera produce auxins, highlighting the genera *Flavobacterium*, *Arachidicoccus*, *Chitinophaga*, *Pedobacter*, *Sphingobacterium* and *Mucilaginibacter* [134,171,172,173,174,175]. Strong evidence supports the IAA role in promoting plant growth and yield by Bacteroidota. While Inoculation with IAA-producing *C. culicis* in barley produced a more robust root system, inoculation with their non-IAA-producing mutants did not. Similarly, *C. culicis* increased the number of spikes in barley field experiments, resulting in higher crop yield [168].

One of the most studied genera within this phylum regarding IAA production is *Flavobacterium*. *Flavobacterium* sp. 72 was able to increase the number of coleoptiles in wheat plants through the production of a total of 1.4 µg mL^−1^ of indole-derived compounds. Of this amount, 1.3 µg mL^−1^ corresponded to indole-3-carboxylic acid, whereas indole-3-lactic acid representing 2.7% of the total production. These results indicate that this bacterium not only produces the main phytohormone involved in plant growth (IAA), but also its derivatives, highlighting alternative plant growth–promoting pathways within members of the phylum Bacteroidota [176]. Other studies using a culture-dependent approach revealed that *Chryseobacterium* sp. NGB-29 and *Flavobacterium* sp. NGB-31 had the most significant effects on promoting maize plant growth, as indicated by both fresh and dry biomass, when compared to PGPRs from the Pseudomonadota group including *Achromobacter*, *Agrobacterium*, *Bordetella*, *Cupriavidus*, *Ochrobactrum*, *Pseudoxanthomonas*, and *Stenotrophomonas*. This effect is explained by their higher IAA production compared with the other bacteria evaluated, in conjunction with nitrogen fixing [177].

Although bacteria of the genus *Flavobacterium* associated with plants are able to synthesize different IAA derivatives, they primarily produce IAA using tryptophan as a precursor. This represents the most widely known IAA biosynthetic pathway in bacteria and is present in up to 82.2% of plant root–associated bacterial strains [178]. The bacterium *Flavobacterium* sp. 11 has been reported to exhibit tryptophan-dependent IAA production regulated by the gene encoding indole-3-glycerol phosphate synthase (*trpC*), which catalyzes the conversion of 1-(o-carboxyphenylamino)-1-deoxyribulose-5-phosphate to indole-3-glycerol phosphate [179].

The same gene has also been reported to be involved in IAA production in *C. culicis*, in which the indole-3-pyruvic acid (IPyA) pathway has been identified as the main metabolic route for IAA biosynthesis [160]. Additionally, the species *Chitinophaga japonensis* BIP-9 has been reported as an IAA producer; however, its inoculation did not promote the growth of *Sorghum* seedlings [180]. This suggests that IAA production is not the only mechanism involved in plant growth promotion, nor the only phytohormone produced by bacteria belonging to the phylum Bacteroidota.

In this regard, the inoculation of *Arabidopsis thaliana* with *Flavobacterium* sp. HYN0056 or *Flavobacterium* sp. G has been reported to activate the expression of genes involved in the ABA response, but not those associated with the biosynthesis of this phytohormone [181]. To date, no studies have demonstrated the production of ABA by bacteria belonging to the phylum Bacteroidota, leaving an open field for exploration within this group. This is particularly relevant given the diverse roles of this phytohormone in plant development and growth, as well as in seed germination and in the regulation of plant stress responses [182]. Similarly, as in the case of ABA, the production of other phytohormones by members of this phylum has not yet been experimentally demonstrated. This is largely because most studies in this area have focused primarily on IAA. Furthermore, genomic approaches highlighting the potential involvement of Bacteroidota in the regulation of other plant phytohormones, based on the presence of genes associated with their biosynthetic pathways in bacterial genomes, remain scarcely explored. These observations indicate that Bacteroidota exhibit functional strategies that differ from those of other major bacterial phyla involved in plant–microbe interactions. To better contextualize these differences, a comparative overview of key ecological and functional traits across dominant phyla is provided in Table 3.

### 4.4. Bacteroidota Role in Plant Resistance to Biotic and Abiotic Stress

Plants are constantly exposed to a range of abiotic stresses (e.g., drought, salinity, temperature fluctuations, nutrient limitations) and biotic stresses (e.g., bacteria, fungi, oomycetes, nematodes, insects) that can negatively impact growth and yield [217,218,219]. Being sessile organisms, plants rely on intrinsic defense mechanisms—largely mediated by phytohormones such as ABA, IAA, brassinosteroids, CKs, ethylene, GA, jasmonic acid (JA), salicylic acid (SA), and strigolactones—to perceive and respond to environmental cues [219,220]. Among these, ABA, SA, JA, and ET are particularly critical in mediating plant defense against pathogens and in conferring tolerance to abiotic stress [221,222,223]. In addition to their intrinsic defenses, plants frequently rely on associations with beneficial microorganisms to withstand those stresses [19,218]. Among these beneficial microorganisms are the Bacteroidota.

#### 4.4.1. Abiotic Stress Tolerance

Ethylene is a key phytohormone involved in plant responses to multiple stresses, including drought and salt stress, and to defense responses to pathogens, but it suppresses plant growth above a certain concentration threshold [224]. Ethylene is synthesized in plants from 1-aminocyclopropane-1-carboxylic acid (ACC); PGPRs effectively enhance plant growth by actively lowering ethylene levels through the deamination of ACC, by ACC deaminase [23,225]. It has been reported that several Bacteroidota genera, such as *Flavobacterium*, *Chryseobacterium*, *Sphingobacterium*, and *Arachidicoccus*, have deaminase activity [139,209,210,226]. Although no studies clearly show that Bacteriodota promote plant growth through this mechanism today, it has been shown that mutants of *Flavobacterium* sp. OR306 expressing heterologous ACC deaminase activity reduced ethylene stress in tomato plants exposed to low-temperature stress, further underscoring their stress-mitigating capacity [167].

In drought-stressed contexts, inoculation with *F. crocinum* HYN0056T upregulated drought-responsive marker genes *RD29A* and *RAB18* in *Arabidopsis thaliana*, thereby enhancing survival through stomatal closure and lateral root development [227]. More recently, *Flavobacterium* sp. GJW24 was shown to promote drought and salt tolerance in both *Arabidopsis* and *Brassica campestris* by upregulating genes associated with root system architecture and stress adaptation [228]. Similar protective effects have been reported in *T. aestivum*, where inoculation improved plant water status, osmolyte accumulation, membrane integrity, and expression of stress-related genes such as *DREB2A* and *CAT1* [229].

#### 4.4.2. Biotic Stress Protection

Members of the phylum Bacteroidota are increasingly recognized for their contribution to plant disease suppression through multiple, complementary mechanisms operating at both strain and community levels. Their antagonistic activity is largely mediated by the production of bioactive metabolites, volatile organic compounds (VOCs), and lytic enzymes, as well as by competitive colonization of plant-associated niches. Several strains have demonstrated effective control of bacterial, fungal and oomycete pathogens in planta, including *P. syringae*, *Phytophthora capsici*, *Colletotrichum musae*, *Lasiodiplodia theobromae*, and *Rhizoctonia solani* [230,231,232,233]. Antagonism has been associated with the production of diffusible and volatile metabolites such as 5,6-dimethylbenzimidazole and 2,4-di-tert-butylphenol, as well as broad-spectrum VOCs that suppress pathogens including *Aspergillus flavus*, *Fusarium graminearum*, *Alternaria alternata*, *Colletotrichum* spp., and *Botrytis cinerea* [231,233,234]. In addition, peptide antibiotics such as chitinocin exhibit broad antibacterial and antifungal activity and significantly reduce disease development under plant conditions [235].

Enzymatic degradation of pathogen structural components represents another key mechanism. Extracellular chitinases produced by Bacteroidota inhibit fungal pathogens such as *F. oxysporum*, *A. alternata*, and *Cladosporium* spp., reduce postharvest disease incidence, and may also affect soilborne pests such as *Meloidogyne incognita* [236]. Genomic analyses further reveal the presence of biosynthetic gene clusters encoding nonribosomal peptide synthetases (NRPSs) and polyketide synthases (PKSs), supporting a strong metabolic potential for secondary metabolite production [73].

Beyond single-strain effects, Bacteroidota are consistently associated with disease-suppressive soils. Increases in Flavobacteriace correlate with suppressiveness against *Ralstonia solanacearum* in tomato [237], and Chitinophagaceae abundance correlates with suppressiveness against *Bipolaris sorokiniana* in wheat [238], while microbiome restructuring through soil fumigation and organic amendments promotes Bacteroidota taxa concomitant with reductions in *R. solanacearum* and bacterial wilt incidence [239]. In sugar beet, disease-suppressive soils challenged with *R. solani* were enriched in Bacteroidota harboring genes for chitinases, NRPSs, and PKSs; functional validation using a synthetic community including *Chitinophaga* and *Flavobacterium* isolates, demonstrated that an NRPS–PKS cluster was essential for fungal suppression [73].

Collectively, these findings indicate that Bacteroidota-mediated disease control relies on an integrated framework combining metabolite production, enzymatic lysis, ecological competition, and microbiome-level modulation, highlighting the potential of Bacteroidota as key contributors to sustainable soil- and plant-based disease management systems.

**Figure 4:**
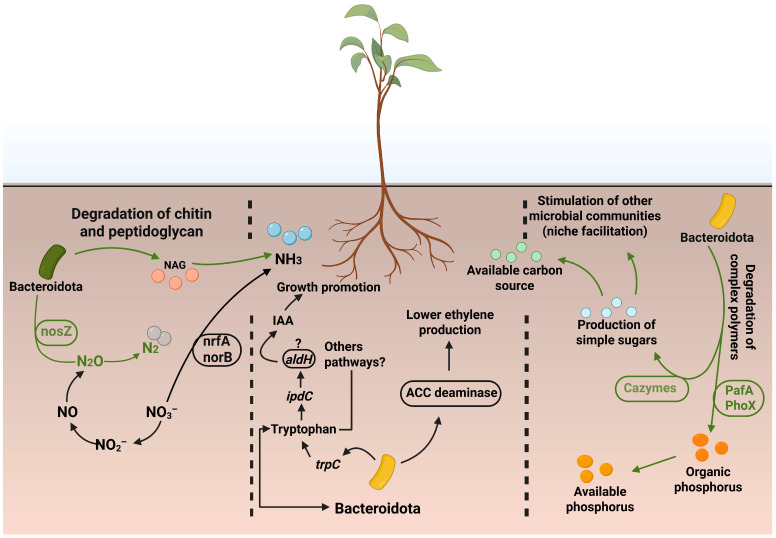
Integrated conceptual model of Bacteroidota-mediated processes in the rhizosphere. Green indicates mechanisms prevalent in Bacteroidota, whereas black denotes processes not universally conserved within the phylum. The model integrates carbon cycling through the initial degradation of complex polymers by carbohydrate-active enzymes (CAZymes), releasing simple sugars and organophosphorylated compounds. These compounds are further mineralized by phosphatases (e.g., PafA and PhoX), liberating inorganic phosphate. CAZymes such as chitinases help inhibit pathogens and release low-molecular-weight compounds that promote beneficial bacteria through the “niche facilitation” effect. In nitrogen cycling, the reduction of nitrous oxide (N_2_O) to dinitrogen (N_2_) is catalyzed by nitrous oxide reductase encoded by the *nosZ* gene. In Bacteroidota, nosZ often occurs without a complete denitrification pathway, indicating a predominant role as N_2_O sinks rather than complete denitrifiers. Complete reduction of nitrate (NO_3_^−^) to N_2_ requires the full denitrification pathway (*nar*/*nap*, *nirK*/*nirS*, *nor*, *nosZ*), which is uncommon in this phylum. Additionally, chitin degradation by chitinases releases N-acetylglucosamine, which can be further metabolized to ammonia (NH_3_). Bacteroidota may also promote plant growth through the production of indole-3-acetic acid (IAA) via tryptophan-dependent pathways (e.g., *ipdC* and *aldH*), although alternative or unknown pathways may also be involved (indicated by “?”). A lower prevalence of ACC deaminase activity suggests a limited role in ethylene modulation.

Finally, this multifunctional strategy (increased nutrient availability, promotion of beneficial bacterial populations, production of plant growth regulators, and control of biotic and abiotic stresses) underscores the importance of Bacteroidota in plant health and fitness (Figure 4).

## 5. Future Prospects and Conclusions

The intensification of agriculture to meet global food demand has traditionally relied on chemical fertilizers and pesticides, yet these inputs are increasingly unsustainable due to their ecological and health impacts. Beneficial microorganisms represent a promising alternative, and while phyla such as Pseudomonadota, Actinomycetota, and Bacillota have been extensively used as biofertilizers and biocontrol agents, Bacteroidota remain comparatively underutilized. Despite their consistent abundance in plant-associated microbiomes, their application in agriculture is limited by challenges in cultivation, isolation, and functional characterization.

Advances in omics technologies including culturomics, metagenomics, transcriptomics, proteomics, and metabolomics are beginning to reveal the ecological significance of Bacteroidota in nutrient cycling, plant growth promotion, and stress tolerance. Some pioneering work has demonstrated the potential of in situ metatranscriptomic profiling of active Bacteroidota to study nitrogen cycle characteristics [126], underscoring the need to extend these approaches to examine their biocontrol characteristics and their participation in the phosphorus cycle. The availability of more complete and high-quality genomes, coupled with improved cultivation techniques such as high-throughput culturomics and microfluidics, will be crucial for unlocking their metabolic potential. In fact, the abundance of the Bacteroidota community in soil has been proposed as an indicator of soil quality, since low abundance of Bacteroidota has been related to poor fertility, while increased abundance of Bacteroidota is also related to good crop yields [240]. In parallel, synthetic community (SynCom) approaches offer a promising strategy to assess synergistic interactions between Bacteroidota and other beneficial taxa under controlled and field conditions.

Future research should prioritize:

1. Expanding genomic resources for Bacteroidota to better characterize their functional diversity and ecological adaptations.
2. Optimizing cultivation strategies, including novel media formulations and in situ cultivation devices, to improve isolation success rates.
3. Integrating multi-omics approaches to identify genes, metabolites, and pathways involved in plant–microbe interactions.
4. Evaluating field performance of Bacteroidota-based inoculants across diverse soils, climates, and cropping systems.
5. Exploring biotechnological applications, including bioremediation of pollutants and enhancement of soil fertility through nutrient recycling.

From a biosafety and regulatory perspective, the potential use of Bacteroidota as bioinoculants requires careful evaluation. Although members of this phylum are widely distributed in soil and plant-associated microbiomes, their pathogenic potential has not been extensively characterized. To date, most reported pathogenic representatives of Bacteroidota are associated with animal hosts, particularly aquatic organisms such as fish (e.g., *Flavobacterium* spp.) [241], as well as occasional opportunistic infections in humans [242]. In contrast, there is currently no clear evidence supporting a significant role of Bacteroidota as plant pathogens.

However, this apparent low risk should be interpreted with caution, as systematic assessments of virulence, host specificity, and environmental safety remain limited. This underscores the need to prioritize biosafety evaluations, including genomic screening and ecological risk assessment, before their use as agricultural bioinoculants.

In conclusion, Bacteroidota represent an underexplored and largely untapped microbial resource for sustainable agriculture. Although their direct use as commercial bioinoculants remains limited, their metabolic versatility particularly in the degradation of complex polymers and nutrient turnover and their influence on plant-associated microbial communities highlight their potential in next-generation, microbiome-based agricultural strategies. Bridging the gap between laboratory findings and field applications will require interdisciplinary efforts integrating microbial ecology, genomics, agronomy, and biotechnology. Addressing these challenges will be essential to harness their potential for improving plant productivity and resilience in sustainable agricultural systems.

## Figures and Tables

**Table 1:** Strategies followed for the isolation of Bacteroidotas from the soil and plant tissues.

Source/Sample	Process	Genus and (Family) Isolated	Reference
Arctic soil (Canada, Norway)	Soil suspension, serial dilution, plating on R2A agar	*Pedobacter* (Sphingobacteriaceae)	[69]
Shore soil (LungmuCo Lake, Tibet, China)	Soil suspension, serial dilution, cultivation on Marine 2216 and R2A media (±NaCl)	*Parapedobacter* (Sphingobacteriaceae)	[70]
Soil (Dokdo Island, Republic of Korea)	Serial dilution and cultivation on nutrient agar	*Flavobacterium* (Flavobacteriaceae)	[71]
Submerged leaf (*Acer palmatum*, freshwater stream, Republic of Korea)	Leaf fragment isolation and cultivation on R2A agar	*Spirosoma* (Cytophagaceae)	[72]
Root endosphere (*Beta vulgaris*, The Netherlands)	Endosphere isolation and cultivation on LB and TSA media	*Chitinophaga* (Chitinophagaceae)	[73]
		*Flavobacterium* (Flavobacteriaceae)	
Onion rhizospheres	Serial dilution and plating on R2A with selective agents (cycloheximide, tobramycin, NaCl variants)	*Flavobacterium* (Flavobacteriaceae) *Chryseobacterium* (Weeksellaceae)	[74]
		*Niabella*, *Taibaiella*, *Flavitalea*, *Chitinophaga* (Chitinophagaceae)	
		*Dyadobacter* (Spirosomataceae)	
Endophytic bacteria (*Dendrobium* roots; ikaite tufa columns, SW Greenland)	Surface-sterilized root tissue—Homogenization and serial dilution; cultivation on TSA- and R2A-derived media (various formulations)	*Ohtaekwangia* (Fulvivirgaceae)	[75]
		*Asinibacterium*, *Niastella*, *Heliimonas* (Chitinophagaceae)	
Ikaite tufa columns (Ikka Fjord, SW Greenland)	Environmental sample, dilution and cultivation on 0.1× R2A agar	*Rhodonellum* (Cytophagaceae)	[76]
Pea and corn root endosphere/rhizosphere (USA)	High-throughput dilution-to-extinction culturomics in low-nutrient (10% TSB) medium	*Flavobacterium* (Flavobacteriaceae), *Chitinophaga* (Chitinophagaceae), *Sphingobacterium* (Sphingobacteriaceae)	[62]
Maize and sunflower endosphere (roots and leaves)	High-throughput dilution-to-extinction culturomics in low-nutrient (10% TSB) medium	*Flavobacterium* (Flavobacteriaceae) *Chitinophaga* (Chitinophagaceae) *Pedobacter* (Sphingobacteriaceae) *Sphingobacterium* (Sphingobacteriaceae)	[67]
Rhizosphere soil (Sinai Desert, Egypt)	Culturomics using serial dilution and cultivation on multiple media (R2A, TSA, MM, TP, TRT, RT)	*Chitinophaga* (Chitinophagaceae) *Niastella* (Chitinophagaceae) *Sphingobacterium* (Sphingobacteriaceae) *Pontibacter* (Hymenobacteraceae)	[77]
Rhizosphere soil (*A. cochinchinensis*)	High-throughput culturomics on multiple media (TSB, LB, Nutrient Broth)	*Flavobacterium* (Flavobacteriaceae)	[78]
Rhizosphere and non-rhizosphere soils of rice (*Oryza sativa* subsp. *japonica*, China)	Culturomics on multiple media (R2A, LB, Ashby)	*Flavobacterium* (Flavobacteriaceae) *Chitinophaga* (Chitinophagaceae) *Pontibacter* (Cytophagaceae) *Sphingobacterium* (Sphingobacteriaceae)	[79]

**Table 2:** Plant growth promotion features from representative bacteroidotas.

Species	Gene	Function	Level of Evidence	Reference
*Chryseobacterium* spp.	*ntrC* *amtB* *nark*, *nrtB*, *nrtC* *nasD*, *nasF*, *nirK* *norB* *fixJ*, *nifA*	Nitrogen metabolism Ammonium transporter Nitrate and nitrite transporter Nitrite reduction Nitrate oxide reduction Nitrogen fixation	Genomic/in silico evidence	[164]
	*cysA* *cysD*, *cysH*, *cysI*, *cysJ*, *cysN*	Sulfate transporter Sulfur metabolism		
	*pstB* *ppK*	High-affinity phosphate transporter Polyphosphate metabolism		
	*zitB zntA*	Zinc transporter		
*Asinibacterium* spp.	*narB*	Ferredoxin nitrate reductase	Controlled functional experimental evidence	[165]
	*nirBD*	Nitrite reductase		
	*nosZLDFY*	Nitrous oxide reductase		
*Flavobacterium* *johnsoniae*	*pafA*	Phosphatase activity (Remineralization of phosphate)	Mechanistic plus environmental functional evidence	[145]
	*phoA1/phoA2*	Classical alkaline phosphatases; Pi-sensitive; contribute to inducible PME activity under Pi limitation		
	*phoX*	PhoX-like lipoprotein; Pi-sensitive; inducible under Pi limitation; may require cofactor for activity		
*Cytophaga hutchinsonii*	*cel9A*, *cel9B*, *cel9C*, *cel5A*, *cel5B*, *bglA*, *bglB*	Genes involved in cellulose degradation, encoding periplasmic or membrane-associated endoglucanases and β-glucosidases, essential for efficient cellulose degradation.	In vitro functional evidence	[166]
*F. johnsoniae*	*gldK*, *gldL*, *gldM*, *gldN*, *sprA*, *sprE*, and *sprT*,	Type IX secretion system (T9SS). Essential for secretion of proteins and gliding motility	Mechanistic/causal evidence	[152]
	*chiA*	Soluble chitinase secreted via T9SS		
*Flavobacterium* sp.	*acdS*	1-aminocyclopropane-1-carboxylate deaminase (ACCD)	Mechanistic/causal validation	[167]
*Chryseobacterium culicis*	*trpC*	Indole-3-glycerol phosphate synthase (tryptophan biosynthesis, IAA precursor)	Field-validated mechanistic evidence	[168]

**Table 3:** Functional differentiation of major bacterial phyla in agricultural contexts.

Characteristics	Bacteroidota	Pseudomonadota	Actinomycetota	Bacillota
Decomposition of organic matter	Degraders of complex plant polysaccharides (e.g., hemicellulose, pectin) with CAZyme diversity; key in intermediate decomposition and cross-feeding [70,71,80,81].	Copiotrophs using labile organic matter; early colonizers and degraders of diverse carbon compounds, including aromatics [183,184,185].	Degraders of recalcitrant organic matter (cellulose, chitin, lignin) via extracellular enzymes [186,187,188].	Copiotrophic and stress-tolerant degraders involved in rapid substrate turnover [189,190].
Nitrogen fixation, and ammonium release	Indirect role in nitrogen cycling; evidence for DNRA and organic N turnover (nosZ clade II) is limited but emerging [123,124,125,126,191].	Central to biological nitrogen fixation in agricultural systems [192,193].	Nitrogen fixation Important in specific ecosystems but less dominant in agricultural soils [194,195].	Minor to moderate diazotrophic potential, mainly in niche environments such as anoxic or organic-rich soils [196,197].
Phosphorus mobilization	Organic P mineralization via phosphatases (PafA, PhoX, PhoA); Pi-regulated or constitutive activity [142,143,144,145].	Main mineral phosphate solubilization via acidification; also, organic P mineralization [198,199].	Predominantly organic P mineralization via extracellular phosphatases; limited mineral phosphate solubilization [199,200].	Mineral phosphate solubilization and organic P mineralization via organic acids and phosphatases [198,199].
Root colonization mechanisms	T9SS, gliding motility surface adhesins [100,151,152,153].	Active swimming and chemotaxis [201].	Filamentous growth (hyphal colonization) [202,203].	Spore production, flagellar motility and biofilm formation [201,204].
Phytohormone production	Indole-3-acetic acid (IAA, tryptophan dependent); limited reports of cytokinins and gibberellin (GA) production [134,171,172,173,174,175]	IAA, cytokinins gibberellins (GA) Abscisic acid (ABA) or ABA-like modulation (rare/indirect reports) [205,206].	IAA, cytokinins, GA [207].	IAA, cytokinins, GA [208].
ACC deaminase	Limited/occasional occurrence [139,209,210].	Dominant strategy [211].	Enriched tendency [212,213].	Frequent [209,211].
Siderophore production	Limited/occasional occurrence [214].	Dominant strategy (Catecholates and hydroxamates) [214,215].	Enriched tendency (Hydroxamates) [214,216]	Enriched tendency (Catecholates) [214,215].
Commercial maturity level	Emerging (underexplored in bioproducts)	Proradix^®^, BioYield (*Pseudomonas* spp.) Serenade^®^, Double Nickel^®^, Subtilex^®^	Actinovate^®^, Mycostop^®^ (*Streptomyces* spp.)	Serenade^®^, Double Nickel^®^, Subtilex^®^ (*Bacillus* spp.)

## Data Availability

No new data were generated in this study. Data supporting the findings of this study are available from publicly accessible databases as cited in the manuscript.
